# The (potential) role of apolipoproteins in nasal mucus of allergic rhinitis patients

**DOI:** 10.1186/2045-7022-4-S2-P19

**Published:** 2014-03-17

**Authors:** Peter Tomazic, Ruth Birner-Grünberger, Obrist Britta, Stefan Spörk, Anita Leitner, Doris Lang-Loidolt

**Affiliations:** 1 ENT-University Hospital Graz, Auenbruggerplatz 26, Graz, 8036, Austria; 2 Medical University of Graz, Institute of Pathology/Center for Medical Research, Graz, Austria; 3 Medical University of Graz, Center of Medical Research, Graz, Austria; 4 Medical University of Graz, ENT-University Hospital Graz, Graz, Austria

## Introduction

Nasal mucus is the first line defense barrier against various allergens. Proteins are functional molecules in mucus and have different roles especially in immune response. Apolipoproteins are plasma proteins that are mainly involved in lipid metabolism, but also have anti-inflammatory properties. The role of this study was to identify apolipoproteins in nasal mucus of allergic rhinitis patients and healthy controls.

## Methods

Fifty-eight individuals (31 male, 27 female) were included in this study. Mean age was 34 years (range: 20-58 years) comprising 29 (50%) allergic rhinitis patients and 29 (50%) healthy controls. Allergy status was verified by skin prick tests and specific IgE. Mucus was taken using a special suction device with a mucus trap and samples were sent for (LC MS/MS) mass spectrometry. Spectral counts (SC) were then obtained and experimental spectra were compared with public databases.

## Results

Apolipoprotein A-IV, Apolipoprotein B-100 were newly identified in nasal mucus and were exclusively present in allergic rhinitis patients not reaching significance. Apolipoprotein A-1 (mean SC=19 vs. 6) and A-2 (mean SC=9 vs. 1) were significantly more abundant in allergic rhinitis patients than healthy controls (3.2 fold and 9.7 fold respectively). Other significantly more abundant proteins were Alpha-2-macroglobulin, Alpha1 antitrypsin and Complement C3.

## Conclusion

Since apolipoproteins were found to have antimicrobial activities and anti-inflammatory properties their significant abundance of Apolipoproteins A-1 and A-2 as well as the presence of Apolipoprotein A-IV and Apolipoprotein B-100 reflect the inflammatory state of allergic rhinitis mucosa and mucus. Moreover they are a marker of increased plasma exsudation into nasal mucus and increased epithelial permeability. The corresponding changes in plasma concentration needs to be determined. This knowledge, however, could serve as an additional marker of severity of inflammation through simple blood samples in allergic rhinitis patients and a plausible local substitution of apolipoproteins could be the target of new anti-inflammatory drugs.

